# Detection of annexin A8 antibodies in serum of patients with antiphospholipid syndrome

**DOI:** 10.11613/BM.2018.030703

**Published:** 2018-10-15

**Authors:** Philipp Scholz, Markus Auler, Johannes Ruthard, Bent Brachvogel, Andreas R. Klatt, Thomas Streichert

**Affiliations:** 1 Institute for Clinical Chemistry, Medical Faculty, University of Cologne, Cologne, Germany; 2 Center for Biochemistry, Medical Faculty, University of Cologne, Cologne, Germany; 3 Department of Pediatrics and Adolescent Medicine, Experimental Neonatology, Medical Faculty, University of Cologne, Cologne, Germany

**Keywords:** annexin A8, antiphospholipid syndrome, antiphospholipid antibodies

## Abstract

**Introduction:**

Antibodies specific for annexin A8 (AnxA8) have not been investigated in patients suffering from antiphospholipid syndrome (APS) yet. The aim of this study was to compare the presence of AnxA8 antibodies in serum of APS patients with that of age-matched healthy controls and to investigate whether AnxA8 antibodies are potential biomarkers for APS.

**Materials and methods:**

We enrolled 22 APS patients and 22 healthy controls in this case-control study. We used sodium dodecyl sulfate polyacrylamide gel electrophoresis and immunoblot to investigate the presence of AnxA8 antibodies, and we applied enzyme-linked immunosorbent assay to investigate the presence of cardiolipin (CL) and beta-2-glycoprotein I (ß2GPI) antibodies.

**Results:**

The serum of 9/22 APS patients showed AnxA8 IgG isotype antibody reactivity compared to serum of 2/22 healthy controls (P = 0.034). When we also included weak immunoblot signals, 12/22 APS patients exhibited AnxA8 IgG isotype antibody reactivity compared to 3/22 healthy controls (P = 0.005). We also investigated the presence of AnxA8 IgM isotype antibodies in the serum of APS patients but found no statistically significant difference between the APS patient group and healthy control group (P = 0.500). We further investigated the presence of ß2GPI and CL IgG and IgM isotype antibodies. AnxA8 IgG isotype antibodies were present in APS patients in a similar frequency as the APS “criteria” antibody against CL (P = 0.764).

**Conclusion:**

We demonstrated that AnxA8 IgG isotype antibodies are potential biomarkers for the diagnosis of APS.

## Introduction

Antiphospholipid syndrome (APS) is an autoimmune disorder that is clinically characterized by thrombosis and/or obstetric complications (*1*-*3*). Antiphospholipid syndrome can occur alone (primary APS) or with other autoimmune diseases (secondary APS), *e.g.*, systemic lupus erythematosus (SLE) (*4*). Due to the lack of specificity in clinical manifestations, the diagnosis of APS is based on the occurrence of clinical symptoms and the detection of at least one of the three antiphospholipid antibodies (aPL, “criteria” aPLs), *i.e.*, IgG or IgM isotype antibodies directed against β2-glycoprotein I (aß2GPI) and cardiolipin (aCL), or a positive lupus anticoagulant (LA) functional assay. Patients diagnosed with APS are placed on lifelong anticoagulation, which is associated with a risk of bleeding complications. Antiphospholipid antibodies titers are used for diagnosis of APS according to the revised Sapporo criteria (> 40 IgM phospholipid units [MPL] or > 40 IgG phospholipid units [GPL]; here, one unit is defined as one microgram of antibody *per* milliliter or > 99th percentile for aCL and > 99th percentile for anti-β2GPI) (*3*, *5*). These criteria also require the presence of aPL on two occasions, 12 weeks apart, to avoid misdiagnosing APS in patients with a low titer or transient aPL (*6*). Laboratory testing is important not only for the diagnosis of APS, but also for risk assessment. Lupus anticoagulant assay is a stronger predictor of risk for vascular thrombosis compared to aCL or aß2GPI, but the greatest risk of thrombosis is found in people with multiple aPLs (*7*). Antiphospholipid antibodies are directed against a heterogeneous group of antigens, *e.g.*, negatively charged molecules, proteins, or phospholipid-protein complexes. Besides the well investigated three “criteria” aPLs, a growing number of “non-criteria” antibodies against various biomolecules, such as prothrombin/phosphatidylserine, vimentin/cardiolipin, protein S, protein C, annexin A2 (AnxA2), annexin A5 (AnxA5), oxidized low-density lipoproteins, lysobisphosphatidic acid, and sulfatides, have been linked to the occurrence of APS (*7*). These “non-criteria” aPLs have been proposed as relevant in APS and useful to subclassify APS with clinical manifestations (*8*, *9*). Therefore, identification of “non-criteria” aPLs is important to assess the risk of APS patients and possibly diagnose patients with APS-like symptoms but without clearly defined laboratory criteria for an APS (seronegative APS, SNAPS).

Annexin A8 (AnxA8) was originally described as an anticoagulant and an inhibitor of phospholipase A_2_ activity due to the 56% association with vascular anticoagulant-alpha (VAC-α, synonyms: AnxA5, lipocortin V) (*10*). In contrast to other annexins, AnxA8 has a low affinity to phosphatidylserine and hardly interacts with the cell surface of dying cells (*11*). Annexin A8 is associated specifically with late endosomes and involved in actin-based late endosome motility (*12*). It is activated by p53 signalling (*13*). Furthermore, AnxA8 may regulate epidermal growth factor receptor signalling and trafficking (*14*). Therefore, prior research speculated that AnxA8 has tumour suppressor effects (*15*). However, the biological function of AnxA8 remains unclear. Recently, the presence of high AnxA8 antibody titers was reported in a patient suffering from SNAPS (*16*). Antibodies specific for AnxA8 have yet not been investigated in patients suffering from APS.

Here, we compared the presence of AnxA8 antibodies in serum of 22 APS patients with that of 22 age-matched healthy controls and investigated whether AnxA8 antibodies are potential biomarkers for APS.

## Materials and methods

This case-control study was performed at the University Hospital of Cologne. We enrolled 22 APS patients and 22 healthy controls. The age of patients in the APS group was 39 (19 - 70) years. Twenty of 22 patients were female. The age of patients in the control group was 41 (19 - 68) years. Five of 22 patients suffered from primary APS, while 17 patients suffered from secondary APS associated with SLE. All APS patients were diagnosed using the revised Sapporo criteria (*3*). The diagnostic criteria require one clinical event, *i.e.*, thrombosis or obstetric complication, and two positive antibody blood tests for one of the “criteria” aPLs 12 weeks apart. All APS patients suffered exclusively from thromboembolic events, except 4 patients who also suffered obstetric manifestations of APS. Patients were excluded if they had other thrombotic risk factors, *e.g.*, factor V-Leiden and prothrombin mutation, antithrombin, protein C and protein S deficiency, and pathological factor VIII and factor XII activity. Remaining samples from routine laboratory testing were used to identify patients for inclusion in the control group. Patients in the control group had no APS symptoms or other autoimmune diseases and no reported thromboembolic or obstetric events in their medical history. Our study was approved by the Ethical Committee of the University Hospital of Cologne (application number 14-176).

Blood samples (remaining samples) were collected from July 2014 to March 2017 in 4.7 mL serum monovettes, centrifuged at 2772xg for 10 minutes, and stored in aliquots at - 70 °C.

We used AnxA8 for the detection of AnxA8 IgG and IgM isotype antibodies in the serum of APS patients. Recombinant expression of AnxA8, sodium dodecyl sulfate polyacrylamide gel electrophoresis (SDS-PAGE), and immunoblot analysis were performed as described previously (*16*). Briefly, AnxA8 was recombinantly expressed in bacteria and purified by affinity- and endotoxin-removal gel chromatography. To investigate the presence of AnxA8 IgG and IgM isotype antibodies in the serum of 22 APS patients and 22 healthy controls, AnxA8 was separated using SDS-PAGE followed by immunoblot analysis with patients’ serum as the primary antibody. The presence of AnxA8 antibodies was visualized by electrochemiluminescence and assessed after 15 and 45 (sensitive assessment) seconds.

Cardiolipin antibodies were assayed with a cardiolipin IgG and IgM enzyme-linked immunosorbent assay (ELISA) using Euroimmun-Analyser I (Euroimmun, Lübeck, Germany), and concentrations of aß2GPI were determined with a Quanta anti-ß2GPI IgG or IgM-ELISA (Inova Diagnostics, San Diego, USA) on a DSX-ELISA-System (Dynex technologies, Chantilly, USA). Both aCL and aß2GPI values are expressed in kU. One kU is the binding activity of 1 mg/l of IgG- or IgM-antibody of the international aCL standard serum (Louisville APL Diagnostics, Seabrook, USA) or the IgG- or IgM aß2GPI reference calibrator (Rheumatology Lab, Seton Hall University, St. Joseph’s Hospital and Medical Center, South Orange, USA), respectively. The cut off of this quantitative method according to the manufacturer is 12 kU for aCL and 20 kU for aß2GPI. According to the manufacturers’ specifications, the coefficient of variation for the anti-cardiolipin assay is 7% and 7.5% for IgG and IgM subtype antibodies and 4.3% and 3.4% for IgG and IgM subtype antibodies of the ß2GPI antibody assay.

P-values, odds ratios, and 95% confidence intervals (95% CI) were calculated using SPSS 22.0.0 (IBM, Chicago, USA). Fisher’s exact test was performed to investigate the relationship between the categorical qualitative data.

## Results

**Figure 1:**
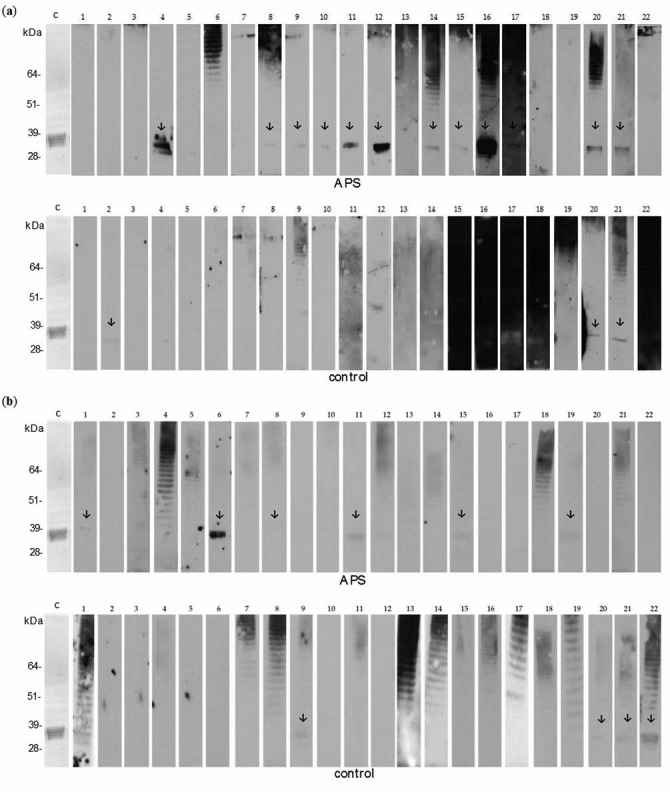
Analysis of annexin A8 (AnxA8) IgG (a) and IgM (b) isotype antibodies in the serum of 22 patients with antiphospholipid syndrome (APS) and 22 healthy controls by SDS-PAGE followed by immunoblot. In (a), the serum of 9/22 APS patients (No. 4, 11, 12, 14, 15, 16, 17, 20, and 21) showed clear AnxA8 IgG isotype antibody reactivity. In addition, serum from patients 8, 9, and 10 showed weak AnxA8 IgG isotype antibody reactivity (sensitive assessment). In contrast, the serum of 2 of 22 (Nos. 20 and 21) or 3 of 22 (Nos. 20, 21, and 2, sensitive assessment) healthy controls were positive for AnxA8 IgG subtype antibodies. Serum of the healthy controls 15, 16, 17, 18, and 22 exhibited a strong background staining but no AnxA8 IgG isotype antibody reactivity. In (b), the serum of 6/22 APS patients (No. 1, 6, 8, 11, 15, and 19) and the serum of 4/22 healthy controls (No. 9 and 20-22) exhibited AnxA8 antibodies. We frequently found serum samples showing IgM isotype antibody reactivity against remaining traces of LPS indicated by a ladder-like pattern (APS No. 4, 18, and 21; control No. 1, 7, 8, 13, 14, 17, 18, 19, and 22). First sample C is showing Coomassie staining of affinity purified AnxA8.

The serum of 9/22 APS patients but only 2/22 healthy controls (P = 0.034) exhibited AnxA8 IgG isotype antibody reactivity (Figure 1a). When we also included weak immunoblot signals (sensitive assessment), 12/22 APS patients exhibited AnxA8 IgG isotype antibody reactivity compared to 3/22 healthy controls (P = 0.005). The odds ratio for APS and the presence of AnxA8 IgG isotype antibodies was 6.9 (95% CI: 1 - 37) and 7.6 (95% CI: 2 - 33) (sensitive assessment).

Six of 22 APS patient sera and 4/22 healthy control sera were positive for AnxA8 IgM antibodies (P = 0.500) (Table 1). Interestingly, in contrast, IgG isotype antibodies were more frequently found in serum samples showing IgM isotype antibody reactivity against remaining traces of lipopolysaccharides (LPS) as indicated by the ladder-like pattern (Figure 1) (*17*).

**Table 1:** Presence of annexin A8 IgG and IgM isotype antibodies in the serum of 22 patients with antiphospholipid syndrome

**AnxA8**	**APS**	**Control**	P*
IgG, N/total	9 / 22	2 / 22	0.034
IgG (s), N/total	12 / 22	3 / 22	0.005
IgM, N/total	6 / 22	4 / 22	0.500
APS - antiphospholipid syndrome. AnxA8 - annexin A8. (s) indicates the results of a sensitive assessment. N/total - number of positive tested persons/total number of participants in group. * Fisher’s exact test, P value < 0.05 was set as a level of statistical significance.			

We further investigated the presence of ß2GPI and CL IgG and IgM isotype antibodies by immunoassay. A functional LA assay was not performed, as APS patients were anticoagulated and anticoagulation could affect the performance of the LA assay. The serum of 10/22 APS patients was positive for aCL IgG isotype antibodies, and the serum of 8/22 APS patients was positive for aCL IgM isotype antibodies. The serum of 9/18 APS patients was positive for ß2GPI IgG isotype antibodies, and the serum of 9/18 APS patients was positive for ß2GPI IgM isotype antibodies. For 4 APS patients, no ß2GPI antibody analysis was available. Frequency of AnxA8 IgG isotype antibodies was similar to frequency of „criteria“ CL antibodies (P = 0.764). Interestingly, one APS patient without CL or ß2GPI antibodies was positive for AnxA8 IgG isotype antibodies.

## Discussion

In this case-control study, we investigated the presence of AnxA8 antibodies in the serum of 22 APS patients and 22 healthy controls. We found a statistically significant difference in the frequency of AnxA8 IgG isotype antibodies between the APS group and the healthy control group. Recently, we reported the case of a SNAPS patient with a history of six pregnancy losses and a fulminant stroke, with no evidence of the three “criteria” aPLs, but with high antibody titers against AnxA2 and AnxA8 (*16*).

Antiphospholipid syndrome diagnosis requires meeting the revised Sapporo criteria of 2006 and detection of at least one of three “criteria” aPLs (*3*). Meanwhile, numerous studies describe the presence of “non-criteria” antibodies in the course of APS. The presence of multiple antibodies is commonly associated with the greatest risk of thrombosis, and increasing interest has been focused on “non-criteria” antibodies (*18*, *19*). The precise relevance of those antibodies and the diagnostic value of a positive test result are unclear, as these antibodies were mostly detected by immunoassays and not by a functional test, as it was described for Annexin A5 anticoagulant activity (*20*). In our study, we used specific immunoblot analysis instead of ELISA analysis to include the molecular size of the detected band as additional selection criteria for the investigation of the serum samples. Several serum samples exhibited antibody reactivity against remaining LPS traces of affinity- and endotoxin-purified AnxA8, which may yield false positive results in ELISA. Analysis by ELISA, therefore, is not suited to discriminate between a specific reactivity against AnxA8 or an unspecific cross-reactivity against remaining traces of LPS.

Prior research on “non-criteria” antibodies was of relatively small sample size, and this is also a limitation of our study (*20*). However, we found a significant difference in the presence of AnxA8 IgG isotype antibodies between the tested groups. Antibodies can occur transiently, particularly IgM isotype antibodies that are linked to infectious disorders. Therefore, persistently elevated aPL levels are a mandatory laboratory criteria for diagnosis of APS, and aPL tests must be repeated within 12 weeks. We investigated the presence of AnxA8 antibodies only once; however, we found a highly significant correlation between the presence of AnxA8 IgG isotype antibodies and APS and identified AnxA8 IgG isotype antibodies as potential biomarkers for the diagnosis of APS.
